# Proton Pump Inhibitors and the Gut Microbiota: From Mechanisms to Clinical Implications

**DOI:** 10.3390/microorganisms14102236

**Published:** 2026-10-03

**Authors:** Franziska Deutschbein, Andrea Severino, Margherita Ferrari, Enrico Capuano, William Fusco, Simone Varca, Debora Rondinella, Tiziano Galasso, Serena Porcari, Antonio Gasbarrini, Gianluca Ianiro

**Affiliations:** 1 Department of Translational Medicine and Surgery, Faculty of Medicine and Surgery, Università Cattolica del Sacro Cuore, 00168 Rome, Italy; franziska.deutschbein@gmail.com (F.D.); andrea.severino@guest.policlinicogemelli.it (A.S.); william.fusco1@guest.policlinicogemelli.it (W.F.); simone.varca@guest.policlinicogemelli.it (S.V.); debora.rondinella@policlinicogemelli.it (D.R.); serena.porcari@policlinicogemelli.it (S.P.); antonio.gasbarrini@unicatt.it (A.G.); 2 UOC CEMAD—Digestive Disease Center, Internal Medicine and Gastroenterology, Department of Medical and Surgical Sciences, Fondazione Policlinico Universitario Agostino Gemelli IRCCS, 00168 Rome, Italy; 3 UOC Medicina Interna e Gastroenterologia—Internal Medicine and Gastroenterology Unit, Department of Medical and Surgical Sciences, Fondazione Policlinico Universitario Agostino Gemelli IRCCS, 00168 Rome, Italy; tiziano.galasso@guest.policlinicogemelli.it

**Keywords:** proton pump inhibitors, gut microbiota, dysbiosis, small intestinal bacterial overgrowth, probiotics

## Abstract

Proton pump inhibitors (PPIs) are among the most widely used drugs in clinical practice. Although generally effective and well tolerated, their widespread and frequently prolonged use has raised concerns about both gastrointestinal and systemic adverse effects. Some of these effects may be directly or indirectly mediated by proton-pump-inhibitor-induced alterations of the gut microbiota. Proton pump inhibitors can reshape gastrointestinal microbial communities through multiple mechanisms, including gastric acid suppression, impaired barrier function, and modulation of the immune and metabolic pathways. The resulting dysbiosis promotes oral-to-gut microbial translocation, reduces microbial diversity, and favors the expansion of potentially pathogenic taxa. These alterations have been associated with several gastrointestinal disorders such as small intestinal bacterial overgrowth, enteric infections, and disturbances of the gut–liver axis, while emerging evidence suggests broader implications for carcinogenesis and the response to immune checkpoint inhibitors. In this context, microbiota-modulating interventions, including probiotics, may provide clinical benefits; however, the current evidence remains limited and inconsistent, precluding their routine use. By integrating mechanistic and clinical evidence, this review aims to clarify the role of the gut microbiota in proton-pump-inhibitor-related adverse effects and to assess the potential of microbiota-targeted strategies to mitigate their clinical consequences.

## 1. Introduction

Since the introduction of Omeprazole in 1989, proton pump inhibitors (PPIs) have become the cornerstone of therapy for acid-related disorders [1]. Their widespread use has placed proton pump inhibitors among the ten most frequently prescribed drug classes worldwide, representing the treatment of choice for a broad range of gastrointestinal conditions, including gastroesophageal reflux disease (GERD), peptic ulcer disease, non-erosive reflux disease, non-steroidal anti-inflammatory drug (NSAID)-associated ulcers, and Zollinger–Ellison syndrome, and as a key component of *Helicobacter pylori* eradication regimens [2,3]. In the general population, approximately 25% of individuals report heartburn at least once per month, thereby contributing to the high demand for proton pump inhibitors [2]. However, proton pump inhibitors are frequently prescribed or self-administered for prolonged periods without a clear evidence-based indication [4,5]. Studies have reported that more than 70% of proton pump inhibitor prescriptions may be inappropriate, with similar patterns observed in hospital settings, where use without a documented indication ranges from 54% to 73% [6,7,8,9,10,11,12,13,14].

Given their extensive use, concerns regarding the long-term safety of proton pump inhibitors have gained increasing attention. Indeed, recent evidence has associated proton pump inhibitors therapy with a broad spectrum of potential adverse outcomes, encompassing both systemic and gastrointestinal effects [15,16,17]. At the systemic level, several studies have reported an increased risk of community-acquired pneumonia among proton pump inhibitors users, particularly during the early phase of treatment [18,19,20]. In addition, prolonged proton pump inhibitor therapy has been linked to impaired absorption of calcium, iron, vitamin B12, and phosphate, with potential consequences for bone health, including an increased risk of fractures [21,22,23]. Emerging evidence has further suggested associations between proton pump inhibitor exposure and neurodegenerative conditions, including dementia [24,25,26]. At the gastrointestinal level, long-term proton pump inhibitor use has been associated with a broad spectrum of adverse outcomes, including small intestinal bacterial overgrowth (SIBO), enteric infections, disturbances of the gut–liver axis, and many other complications [16,27,28].

Against this background, the gut microbiota has emerged as a compelling potential mediator of proton-pump-inhibitor-related adverse effects, drawing increasing attention to how these drugs reshape microbial composition and host–microbiota interactions.

Evidence from next-generation sequencing studies indicates that proton pump inhibitors induce marked changes in the intestinal microbiota, partly due to impaired gastric acid barrier function, thereby facilitating oral-to-gut microbial transmission and distal colonization by upper gastrointestinal and oral microorganisms [16,29,30,31]. Large cohort studies and meta-analyses have associated proton pump inhibitor use with reduced microbial diversity and recurrent taxonomic shifts, although the magnitude of these alterations varies across populations and exposure durations [14,29,30]. Such perturbations have been linked to small intestinal bacterial SIBO [16], enhanced bacterial translocation [32], and hypochlorhydria-related *Enterococcus* overgrowth, potentially exacerbating ethanol-induced liver disease [33].

In light of this evidence, this review critically synthesizes the current knowledge on the mechanisms through which proton pump inhibitors reshape the gut microbiota; it examines how the resulting dysbiosis may contribute to clinically relevant gastrointestinal and systemic outcomes. By integrating mechanistic and clinical evidence, it aims to clarify the role of the gut microbiota in PPI-related adverse effects and to assess the potential of microbiota-targeted strategies to mitigate their clinical consequences.

### Literature Search and Evidence Synthesis

This article was designed as a narrative, non-systematic review. No prospective review protocol was registered, and no formal PRISMA-compliant screening or study-selection workflow was performed. Relevant literature was identified through searches of PubMed, Scopus, and Web of Science, with the final literature search performed on 15 July 2026. Core search concepts included combinations of terms related to “proton pump inhibitors”, “gut microbiota” or “gut microbiome”, “dysbiosis”, “small intestinal bacterial overgrowth”, “enteric infections”, “probiotics”, “cancer”, and “immune checkpoint inhibitors”. Reference lists of relevant reviews and key original articles were also examined to identify additional pertinent studies.

Given the narrative scope of the review, evidence was selected according to its relevance to the mechanistic and clinical questions addressed. Priority was given to human randomized controlled trials, systematic reviews and meta-analyses, large observational cohort studies, and clinical guidelines. Mechanistic in vitro and animal studies were included when they provided biological plausibility or helped clarify pathways that could not be directly investigated in humans. Studies with limited relevance to PPI-associated microbiota alterations, duplicate reports, and publications not providing sufficiently informative mechanistic or clinical data were generally not prioritized.

Because this was not a systematic review, literature retrieval may be subject to literature-selection bias. During evidence synthesis, concordant and conflicting findings were considered together whenever available. Greater interpretative weight was given to higher-level clinical evidence and adequately designed human studies, whereas preclinical findings were used primarily to support mechanistic plausibility rather than to infer clinical causality. Where results were inconsistent, this uncertainty was explicitly acknowledged in the text rather than resolving discrepancies in favor of a single interpretation.

## 2. Proton Pump Inhibitors: Mechanisms of Action and Clinical Indications

### 2.1. Mechanism of Action

Proton pump inhibitors have revolutionized the management of acid-related disorders [34]. Their development followed the era of H_2_-receptor antagonists, which, although effective, provided incomplete acid suppression [35]. A major breakthrough was achieved by targeting the final common pathway of gastric acid secretion: namely, the hydrogen/potassium adenosine triphosphatase (H^+^/K^+^ ATPase) located on the secretory canalicular membrane of gastric parietal cells [3,16]. Early research conducted in the late 1970s led to the identification of precursor compounds such as Picoprazole and Timoprazole, which confirmed the relevance of this therapeutic target despite initial toxicity concerns [36,37,38]. The synthesis of Omeprazole in 1979 marked a pivotal advance and, following its launch in 1988, it became a cornerstone therapy for acid-related disorders, leading to the development of newer agents such as Lansoprazole and Esomeprazole [34].

Proton pump inhibitors exert their antisecretory effect through the targeted, mechanism-based inhibition of gastric acid production [3]. They are administered as inactive, acid-labile prodrugs that, after systemic absorption, selectively accumulate within the acidic compartments of activated parietal cells. Within this environment (pH < 4), they undergo acid-catalyzed rearrangement to form reactive sulfenamide species that covalently bind to cysteine residues on the H^+^/K^+^ ATPase, resulting in irreversible inactivation [3]. This accounts for their prolonged pharmacodynamic effect, with acid suppression lasting up to 24–72 h despite a short plasma half-life, as restoration requires de novo pump synthesis [3].

### 2.2. Clinical Indications

The potent and sustained acid-suppressive effect of proton pump inhibitors underpins their broad clinical use [1]. Among their principal indications, they represent the first-line pharmacological treatment for both erosive and nonerosive GERD: an initial course of four to eight weeks is recommended to achieve symptom control and mucosal healing, followed by dose reduction, intermittent or on-demand therapy, or discontinuation when feasible; conversely, long-term maintenance at the lowest effective dose is generally required in patients with severe erosive esophagitis or other GERD-related complications [37,39]. PPIs are also central to the management of peptic ulcer disease, acid suppression as part of *Helicobacter pylori* eradication regimens, treatment of NSAID-associated ulcers, and high-dose therapy following endoscopic hemostasis of bleeding ulcers [40,41,42]. Sustained, often high-dose PPI therapy remains the standard of care for controlling the marked gastric acid hypersecretion associated with Zollinger–Ellison syndrome [43].

Beyond the treatment of established disease, PPIs are recommended for gastroprotection in patients at increased risk of upper gastrointestinal bleeding who require long-term non-steroidal anti-inflammatory drugs, dual antiplatelet therapy, anticoagulation, or combinations of antithrombotic agents [29,41,44,45]. Relevant risk factors include advanced age, a history of peptic ulcer or gastrointestinal bleeding, and concomitant use of NSAID or corticosteroids; however, corticosteroid or anticoagulant therapy alone does not routinely warrant PPI prophylaxis in the absence of additional gastrointestinal risk factors [44].

## 3. Gut Microbiome and Proton-Pump-Inhibitor-Related Alterations

### 3.1. Gastrointestinal Microbiome

The human gastrointestinal system harbors a highly complex and dynamic microbial ecosystem, commonly referred to as the microbiota, which represents an integral component of the gastroenteric environment and is widely recognized as a key determinant of host health [46]. This ecosystem consists of a highly diverse and dynamic assemblage of microorganisms, including bacteria, archaea, viruses, and fungi, with bacteria representing the predominant component [47]. Within this dynamic system, microbial composition is shaped by a complex interplay of host-related and environmental factors, such as age, diet, genetic background, and environmental exposures [47]. This microbial community is not uniformly distributed but is organized along the length of the digestive tract, where distinct anatomical regions are characterized by markedly different physiological conditions, including variations in pH, oxygen availability, host-derived secretions, and nutrient supply. These spatially distinct environments play a critical role in shaping the composition and functional organization of local microbial communities [48]. Among the microbial communities inhabiting the different compartments of the gastrointestinal tract, the gut microbiota is by far the most extensively characterized and the one with the best-understood functional roles [47].

Functionally, the gut microbiota plays a central role in a broad range of physiological processes essential for host homeostasis. These include the maintenance of colonization resistance against potentially pathogenic microorganisms, the regulation of immune system development and maturation, and the modulation of nutrient absorption and metabolic pathways. In addition, the gut microbiota mediates host interactions with xenobiotic compounds, including pharmacological agents, thereby influencing drug metabolism and bioavailability [46,49,50,51,52]. Beyond these functions, the gut microbiota contributes to the production of a wide array of bioactive metabolites that act as signaling molecules at both local and systemic levels, exerting a significant influence on host physiological homeostasis and overall health [53].

Given its functional relevance and dynamic nature, the gut microbiome’s composition is highly sensitive to a variety of host-related and environmental influences, among which medications represent one of the most impactful. Several commonly prescribed drugs, including antibiotics and PPIs, have been shown to significantly alter microbial community structure and function, thereby increasing susceptibility to a variety of pathological conditions [54].

Drug–microbiota interactions may be bidirectional, as interindividual differences in gut microbial composition and function can also influence drug bioavailability, bioactivity, efficacy, and toxicity through direct microbial metabolism or indirect interactions with host metabolic and immune pathways [55]. However, while this interaction has been demonstrated for several pharmacological agents, evidence regarding PPIs has predominantly focused on their effects on microbial composition. To the best of our knowledge, no specific microbial taxa or strains have yet been identified as determinants or predictors of the PPI therapeutic response. Whether baseline microbial signatures contribute to interindividual variability in PPIs’ efficacy therefore remains an important area for future investigation.

### 3.2. How Do Proton Pump Inhibitors Affect the Gastro-Intestinal Microbiome?

A substantial body of evidence indicates that long term use of PPIs is associated with significant alterations in the composition of the gastrointestinal microbiome [14,29,30,31]. Although microbiome alterations have also been reported following short-term PPI exposure, the nature and extent of these changes have not been consistently reproduced across studies [29,56,57,58,59].

The primary mechanism underlying this effect is the suppression of gastric acid secretion, which disrupts one of the most important physiological barriers limiting microbial survival and colonization along the gastrointestinal tract [14,30]. Reduced gastric acidity permits microorganisms originating from the oral cavity and upper gastrointestinal tract to survive gastric transit and reach more distal intestinal segments, where they may establish persistent colonization [29,31]. Notably, PPIs appear to promote not only the passive survival but also the expansion and distal transmission of specific oral taxa (such as *Fusobacterium nucleatum* and *Streptococcus anginosus*), as demonstrated in a randomized controlled trial, suggesting a mechanism that extends beyond simple acid suppression [57]. Concomitantly, PPIs have been shown to directly inhibit selected commensal gut bacteria, including members of the *Ruminococcus* and *Dorea* genera [29].

Beyond luminal pH modulation, PPIs may influence the gut microbiome through direct and indirect modulation of host physiological processes [29]. While clinical evidence supports the role of gastric acid suppression in promoting microbial survival and translocation, evidence for these additional pathways derives predominantly from in vitro experiments and animal models, with direct clinical evidence remaining relatively limited. Preclinical studies suggest that PPIs may impair epithelial barrier integrity by altering tight junction protein expression, thereby increasing intestinal permeability and facilitating the translocation of bacteria or bacterial products [3,60]. Similarly, experimental evidence indicates that PPIs may interfere with innate immune function, particularly neutrophil and macrophage activity, potentially compromising mucosal defense against pathogenic microorganisms [3,60] Alterations in bile acid metabolism represent another potential pathway through which PPIs shape microbial communities: by modifying the pH of the proximal small intestine, PPIs may influence bile acid solubility and microbial biotransformation, thereby selecting for bile-tolerant taxa and reshaping microbial metabolic activity. However, clinical evidence remains limited, and at least one interventional study found no significant changes in bile acid profiles during PPI therapy [59,61]. Alterations in gastrointestinal motor function may represent an additional, although less thoroughly established, pathway contributing to PPI-associated microbiota perturbations: PPI therapy has been associated with delayed gastric emptying, particularly of solid meals, an effect primarily attributed to impaired acid-dependent peptic digestion, whereas hypergastrinemia appears to play only a minor role [62,63].

Collectively, the current evidence identifies gastric acid suppression and the consequent weakening of the gastric acid barrier as the principal and best-established drivers of PPI-induced microbiota perturbations, supported by evidence from human studies. Direct effects on selected microbial taxa may also contribute to these alterations. In contrast, indirect host-mediated pathways, including changes in epithelial permeability, immune function, bile acid metabolism, and gastrointestinal motility, remain less firmly established and are supported predominantly by preclinical evidence, with direct clinical validation still limited.

However, the available evidence is characterized by substantial heterogeneity in PPI exposure and patient characteristics. PPIs are frequently analyzed as a single pharmacological class, while differences among individual compounds, dosages, treatment intensity, and duration of exposure are not consistently accounted for [55,59]. This is particularly relevant because microbiome alterations may depend not only on the presence of acid suppression but also on its magnitude and duration. Moreover, patient-related factors may further contribute to variability across studies. Underlying comorbidities can independently affect gut microbiota composition and are often closely related to the clinical indication for PPI therapy, introducing potential confounding by indication. Concomitant medications represent an additional source of heterogeneity. Overall, these factors limit direct comparability between studies and currently preclude firm conclusions regarding compound-specific, dose-dependent, or duration-dependent effects of PPIs on the gut microbiota.

### 3.3. Microbiome Alterations Along Different Gastrointestinal Tracts

Within this framework, the impact of these processes is further shaped by local ecological conditions along the gastrointestinal tract, resulting in non-uniform microbial responses across distinct anatomical regions. PPIs exert region-specific effects on microbial communities, largely driven by the overgrowth and distal translocation of oral-origin bacteria—most prominently members of the *Streptococcaceae* family—together with a concomitant reduction in key commensal taxa [29].

At the level of the oral cavity, PPI exposure has been associated with measurable shifts in microbial composition, characterized by an increased relative abundance of *Streptococcus* and a reduction in genera such as *Neisseria* and *Veillonella,* based on the analysis of saliva, periodontal pocket fluid and fecal samples [64].

These changes suggest that PPIs may influence microbial ecology even in upstream compartments, potentially contributing to downstream seeding of the gastrointestinal tract.

Regarding the esophagus, available evidence based on the analysis of esophageal biopsies indicates that PPIs use does not substantially alter overall microbial community structure. However, subtle compositional changes have been reported, including modest increases in *Actinomyces* and *Streptococcus*, suggesting that local environmental modulation may favor the expansion of selected oral-associated taxa without inducing overt dysbiosis [65].

More pronounced alterations are observed in the stomach and proximal small intestine, where suppression of gastric acidity creates a permissive environment for colonization by oral and pharyngeal bacteria. Studies assessing the upper gastrointestinal microbiota through gastric fluid and gastric biopsies have reported increased abundances of *Streptococcus*, *Rothia*, *Staphylococcus*, and *Enterococcus*, alongside a reduction in acid-adapted taxa such as *Helicobacter pylori* in the absence of active infection [31,66].

In the distal intestine, particularly the colon, PPI use is associated with more profound and clinically relevant microbiome perturbations, predominantly characterized through fecal microbiome analysis. These include a reduction in overall microbial diversity and a marked enrichment of oral-origin bacteria, such as *Rothia*, *Streptococcus*, *Staphylococcus*, and *Enterococcus*, as well as potentially pathogenic species including *Escherichia coli* [14,29]. Concurrently, a depletion of beneficial anaerobic commensals, notably members of the *Ruminococcaceae* and *Lachnospiraceae* families, has been observed [29]. Emerging evidence further supports the role of PPIs in facilitating oral-to-gut microbial transmission, with increased detection of oral taxa such as *Fusobacterium nucleatum* and *Streptococcus anginosus* in the colonic microbiota of proton pump inhibitor users [57].

Taken together, these findings indicate that PPI-associated microbial alterations extend throughout the gastrointestinal tract while displaying distinct site-specific patterns.

## 4. Gastrointestinal Adverse Outcomes Related to PPI Use

**Figure 1:**
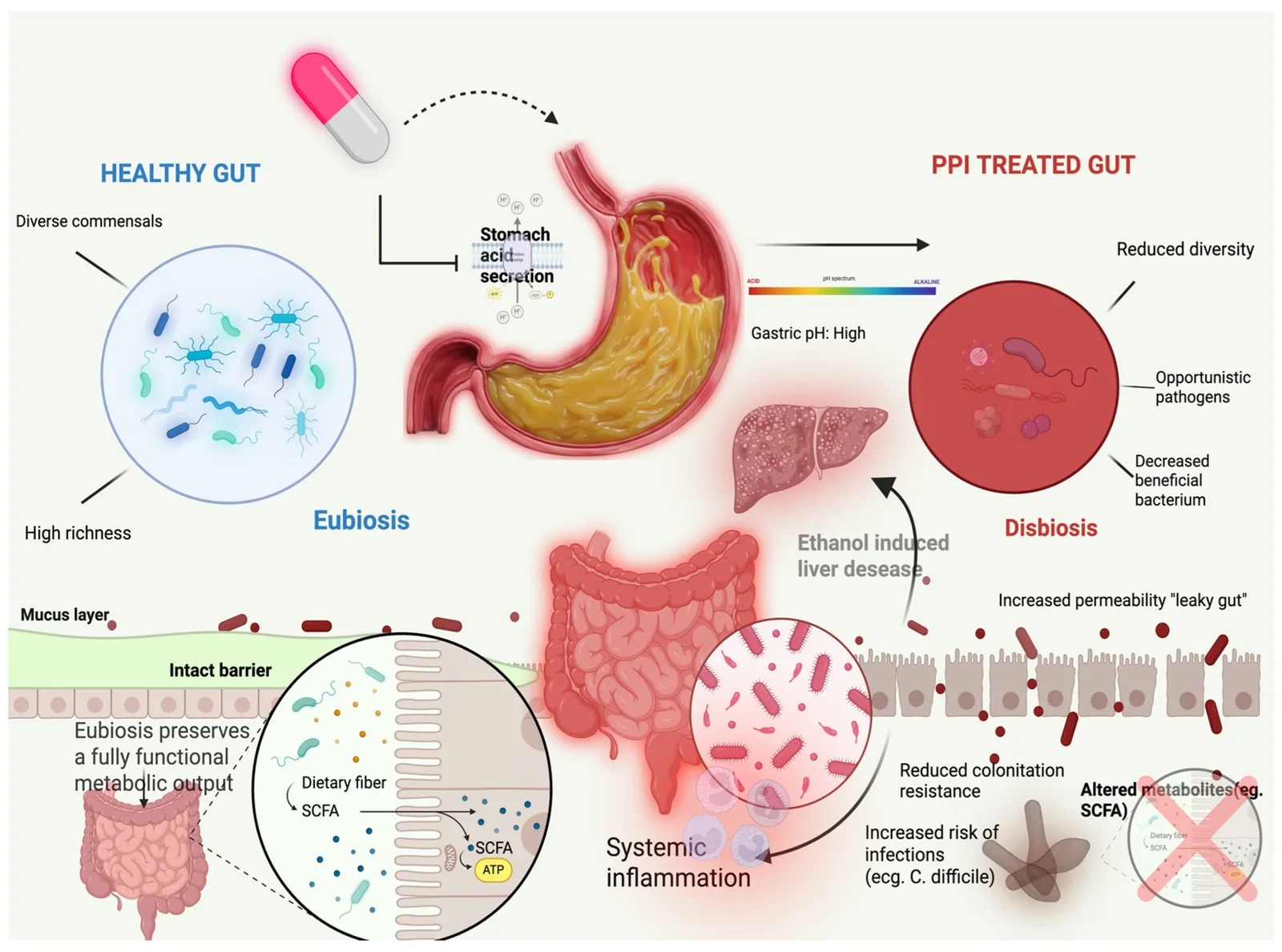
Proton Pump Inhibitors and the Gut Microbiota: From Eubiosis to Dysbiosis. The left side illustrates a healthy intestinal ecosystem, characterized by high microbial richness and diversity, abundant beneficial commensals, an intact epithelial barrier, effective colonization resistance, and balanced microbial metabolite production. The right side depicts the PPI-treated gut: suppression of gastric acid secretion and the resulting increase in gastric pH promote dysbiosis, characterized by reduced microbial diversity, depletion of beneficial bacteria, and expansion of opportunistic microorganisms. These alterations may impair epithelial barrier integrity and colonization resistance, modify microbial metabolite production, and contribute to enteric infections, systemic inflammation, and disturbances of the gut–liver axis. ATP, adenosine triphosphate; PPIs, proton pump inhibitors; SCFAs, short-chain fatty acids. Created in BioRender. Capuano, E. (2026) https://BioRender.com/smqokka.

This dysbiotic pattern, characterized by gastrointestinal “oralization”, depletion of beneficial anaerobic commensals, and reduced colonization resistance, provides a plausible biological framework for a range of gastrointestinal disorders that have been associated with long-term PPI use. However, most of the clinical evidence discussed below derives from observational studies and should therefore be interpreted as associative rather than causal. Residual confounding factors as clinical indication, concomitant medications, underlying comorbidities and reverse causation may contribute to some of the reported associations. Figure 1 schematically illustrates the transition from gastrointestinal eubiosis to PPI-associated dysbiosis and its main local and systemic consequences, while Table 1 summarizes the principal PPI-associated gastrointestinal adverse outcomes, their level of evidence, proposed pathogenetic mechanisms, and main confounders.

### 4.1. Small Intestinal Bacterial Overgrowth (SIBO)

Multiple observational studies, clinical trials and meta-analyses have examined the association between PPI therapy and SIBO. As summarized in Table 1, PPI users show a higher risk of SIBO compared with non-users.

PPI use has been associated with a higher prevalence and increased odds of SIBO compared with non-use, with pooled ORs of 1.71 and 2.14 in separate meta-analyses and estimated prevalences of 36.8% versus 19.9%, respectively [67,68]. Importantly, the magnitude of this association should be interpreted in the light of the diagnostic method used: studies employing duodenal or jejunal aspirate cultures, regarded as the reference standard for SIBO diagnosis, have reported associations as high as 7.6-fold, whereas breath-test-based studies have generally yielded weaker or less consistent findings [68,69]. Notably, in the subgroup of studies using the glucose hydrogen breath test, the association between PPI use and SIBO was not statistically significant (OR 1.93, 95% CI 0.69–5.42), further highlighting the substantial influence of the diagnostic method on the reported association [69].

This association appears to be dependent on duration: although SIBO has been observed in 7.8% of healthy volunteers after only seven days of PPI exposure [66,69], meta-regression analyses indicate an approximately 4% increase in SIBO prevalence for each additional month of treatment [67,70].

Consistent with these findings, the American Gastroenterology Association (AGA) Clinical Practice Update recognizes gastric acid suppression as a predisposing factor for SIBO, while emphasizing the need for further high-quality studies to clarify causality and dose–response relationships [71].

### 4.2. Enteric Infections

Beyond bacterial overgrowth, PPI therapy has been associated with increased susceptibility to infectious complications, particularly enteric and nosocomial infections.

The most consistently reported association concerns *Clostridioides difficile* infection (CDI). Meta-analyses of hospitalized patients show that PPI exposure is linked to an increased risk of hospital-acquired CDI, with pooled odds ratios of approximately 1.8 despite substantial heterogeneity among studies [72].

Conversely, evidence from randomized trials is less supportive of this association. A recent systematic review and meta-analysis of eight RCTs including 29,880 participants found no statistically significant increase in CDI among proton pump inhibitors users compared with non-users (RR 1.19, 95% CI 0.75–1.89), with similarly non-significant results when proton pump inhibitors were compared with H2-receptor antagonists. These findings underscore the potential influence of residual confounding in observational estimates [73].

**Table 1:** Summary of the main adverse outcomes associated with proton pump inhibitor use, including level of evidence, proposed pathogenetic mechanisms, and major confounders and limitations.

Proton Pump Inhibitors Class (If Specifically Associated)	Associated Condition	Study Type	Level of Evidence	Key Bibliographic References	Main Pathogenetic Mechanism	Main Confounders/Limitations
General PPIs	Small intestinal bacterial overgrowth (SIBO)	Review; Meta-analysis; Systematic review and meta-analysis; Prospective study	Moderate-to-high	Tian et al. (2023) [74] Su et al. (2018) [68] Khurmatullina et al. (2025) [67] Durán-Rosas et al. (2024) [70] Quigley et al. (2020) [71]	Gastric acid suppression is the main driver of SIBO. Oral-to-gut microbial translocation (oralization) allows oral taxa to colonize the distal gut. Impaired epithelial barrier increases permeability and bacterial translocation. Immunomodulation reduces bacterial control. Altered bile acid metabolism (uncertain contribution). Delayed gastric emptying causes intestinal stasis.	Diagnostic heterogeneity (duodenal/jejunal aspirate vs breath test); variable exposure duration; possible confounders (motility, GI comorbidities); causality not proven.
General PPIs	Increased risk of enteric infections	Observational cohort study; Systematic review and meta-analysis	Moderate	Chen et al. (2016) [75] Hafiz et al. (2018) [76]	Reduced gastric acidity weakens the host barrier, letting pathogens (like *Salmonella* and *Campylobacter*—the two with the strongest risk) survive gastric transit. Impaired innate immunity, mainly neutrophils and macrophages, weakens mucosal defense. PPI-induced dysbiosis lowers colonization resistance, favoring pathogen establishment.	Confounders (immune status, dietary exposures, travel); possible exposure misclassification bias; not observed with H2RA.
General PPIs	Increased C. difficile infection risk	Umbrella review; Systematic review and dose-response meta-analysis; Review; Meta-analysis	Moderate-to-high	Nalabothula et al. (2025) [77] Finke et al. (2025) [78] Tian et al. (2023) [74] Arriola et al. (2016) [72]	Reduced gastric acidity impairs the host barrier, allowing *C. difficile* to survive gastric transit. Impaired innate immunity, mainly neutrophils and macrophages, weakens mucosal defense. PPI-induced dysbiosis lowers colonization resistance, favoring *C. difficile* establishment. PPIs consistently increase CDI risk (OR 1.26–2.34), highest in ICU and recurrent CDI. PPI dose and duration linearly increase CDI risk (RR 1.02/day; 1.54 at 40 mg), with no safe threshold. PPIs significantly increase hospital-acquired CDI risk (OR 1.81).	High heterogeneity across studies; confounders (hospitalization, antibiotics, age, comorbidities); possible confounding by indication; higher risk in ICU and recurrent CDI.
General PPIs	Systemic infections	In vitro and in vivo study; Cohort study	Moderate	Haas et al. (2018) [79] Lassalle et al. (2023) [80]	Reduced gastric acidity weakens the barrier against ingested pathogens. Impaired innate immunity reduces neutrophil and NK function, promotes M2 macrophages and inhibits lysosomal enzymes. Impaired adaptive immunity suppresses CD4^+^ T cells, reduces Th1/Tregs and increases Th17. Gut dysbiosis and increased permeability facilitate bacterial translocation.	Confounders (type of surgery, pediatric age, comorbidities); direct immune versus microbiota-mediated mechanisms not fully separated.
General PPIs	Increased risk of spontaneous bacterial peritonitis in cirrhotic patients	Observational study	Low-moderate	Vos et al. (2013) [32]	PPIs facilitate intestinal bacterial proliferation by suppressing gastric acid, alter gastrointestinal motility, and impair neutrophil function, thereby promoting bacterial translocation and consequently SBP.	Confounders (cirrhosis severity, ascites, other therapies); limited data.
General PPIs	Malattia epatica alcolica (ALD)/encefalopatia epatica	Experimental mouse model	Low-moderate	Llorente et al. (2017) [33]	PPIs increase intestinal Enterococcus, which translocates to liver, activates TLR2 on Kupffer cells, induces IL-1β, worsening alcoholic liver disease in mice and humans.	Confounders (cirrhosis severity, alcohol consumption, other therapies).
General PPIs	Increased mortality and hepatic decompensation in liver cirrhosis	Observational cohort study	Low-moderate	Roza et al. (2019) [81]	PPIs increase mortality (aHR 2.10) and hepatic decompensation hospitalizations (aRR 1.61) in decompensated cirrhosis;	Confounders (cirrhosis severity, alcohol consumption, other therapies).
General PPIs	Increased mortality in patients with cirrhosis	Meta-analysis of cohort studies	Low-moderate	Wu et al. (2020) [82]	PPIs suppress gastric acid, causing bacterial overgrowth in stomach and intestine; impaired intestinal barrier in cirrhosis facilitates bacterial translocation to mesenteric lymph nodes, leading to SBP and infections; PPIs contribute to gut dysbiosis, and altered microbiota may induce or exacerbate hepatic encephalopathy.	Confounders (cirrhosis severity, alcohol consumption, other therapies).
General PPIs	Increased risk of hepatic encephalopathy in advanced liver disease	Meta-analysis	Low-moderate	Tantai et al. (2019) [83]	PPIs promote hepatic encephalopathy by disrupting the gut-liver axis, with gastric acid suppression causing intestinal *Enterococcus* overgrowth, increased permeability and bacterial translocation to the liver	Confounders (cirrhosis severity, alcohol consumption, other therapies).
General PPIs–higher risk with omeprazole/lansoprazole versus pantoprazole/esomeprazole.	Increased gastric cancer risk	Meta-analysis of cohort studies; Population-based study; Comprehensive review	Moderate	Tran et al. (2024) [84] Brusselaers et al. (2025) [85] Cheung et al. (2018) [86] Sawaid et al. (2024) [87]	PPIs promote gastric cancer by suppressing acid and causing hypergastrinemia, which drives ECL hyperplasia and mucosal changes, while hypochlorhydria alters the gastric microbiome, favoring bacterial overgrowth and N-nitroso compound production, potentially creating a permissive microenvironment for carcinogenesis, including after *H. pylori* eradication.	Confounders (*H. pylori*, atrophic gastritis, intestinal metaplasia); protopathic bias; possible residual confounding;
General PPIs-omeprazole/lansoprazole > pantoprazole/esomeprazole	Hypergastrinemia, ECL cell proliferation, mucosal hyperplasia	Systematic review	Moderate (dose-dependent effect	Lundell et al. (2015) [38]	PPIs cause sustained acid suppression, which induces chronic hypergastrinemia, acting as a trophic stimulus on the gastric mucosa, promoting enterochromaffin-like cell proliferation and mucosal hyperplasia, and potentially contributing to gastric carcinogenesis.	Confounders (*H. pylori*, atrophic gastritis); variable exposure duration.
General PPIs	CRC	Retrospective cohort study; Systematic review and meta-analysis; Observational study	Low (association not consistently reproducible; SIR 1.10 only for right-sided colon and young adults)	Liu et al. (2024) [88] Guo et al. (2023) [89] Abrahami et al. (2022) [90] Ma et al. (2020) [91]	PPIs may promote colorectal cancer through oralization and dysbiosis, enriching CRC-associated bacteria (e.g., *Fusobacterium*, *E. coli*, *Enterococcus*, *Streptococcus*, *Clostridium perfringens* enterotoxin), reducing diversity and colonization resistance, activating YAP and Th17 pathways, and possibly inducing hypergastrinemia, especially right-sided and in young adults.	Confounders (screening, diet, CRC risk factors); heterogeneity across studies; lack of direct longitudinal confirmation; possible protopathic bias.
General PPIs	Inflammatory bowel disease (IBD)	Review; Systematic review and meta-analysis	Low-to-moderate	Tian et al. (2023) [74] Gao et al. (2026) [92]	PPIs may promote IBD by reducing *L. reuteri* and histamine conversion, increasing TNF-α, suppressing gastric acid and altering the gut microbiome, reducing SCFAs, weakening the epithelial barrier and Treg function, increasing permeability and LPS translocation, activating TLR4-NF-κB and Th17 inflammation, with dysbiosis and *C. difficile* triggering relapse.	Confounders (IBD therapies, disease activity, antibiotics); heterogeneity across studies.
General PPIs	Cholelithiasis	Review	Low	Tian et al. (2023) [74]	PPIs may promote cholelithiasis by altering the gut microbiota, increasing BSH-producing *Lactobacillus* and *Clostridium* and 7α-dehydroxylase-producing *Eubacterium*, thereby promoting secondary bile acid formation and gallstone development.	Confounders (diet, obesity, genetic factors); microbiota-mediated mechanisms not confirmed in dedicated clinical studies.
General PPIs	Osteoporosis	Critical reviews	Moderate	Martiniakova et al. (2026) [93]	PPIs may contribute to osteoporosis by decreasing Ruminococcaceae and Lachnospiraceae while increasing *Enterococcus*, *Streptococcus*, *Staphylococcus* and *Rothia*, potentially impairing bone health, alongside altered absorption of calcium, iron, vitamin B12 and phosphate.	Confounders (age, nutritional status, comorbidities); direct mechanisms (malabsorption) versus indirect mechanisms (dysbiosis) are difficult to separate.

In addition to CDI, observational studies report elevated risks of other enteric infections among proton pump inhibitor users. A meta-analysis of observational data found more than a fourfold increased risk of enteric infections overall, with particularly strong associations for *Salmonella* and *Campylobacter* species [75,76]. Large population-based cohorts also demonstrate a dose–response relationship between PPI exposure and hospitalization for infectious gastroenteritis, an association not observed with H2 receptor antagonists [75].

Reduced gastric acidity and impaired immune responses represent biologically plausible mechanisms that may contribute to these observations, as both can compromise host defense mechanisms against ingested pathogens [72,79].

Together, these findings support an association between PPI exposure and susceptibility to enteric infections, while not establishing a direct causal relationship. They also reinforce the importance of careful risk–benefit assessment, particularly in vulnerable populations.

### 4.3. Alcohol-Associated Liver Disease

PPIs use is common among patients with alcohol-associated liver disease (ALD), with approximately 40% of individuals chronically exposed to these medications, often without a clear clinical indication [94].

This high prevalence has raised concerns, as several clinical studies have linked proton pump inhibitor therapy to adverse outcomes in this population. Observational cohorts show that PPI use is associated with increased all-cause mortality in patients with ALD [81,82]; as well as an elevated risk of hepatic encephalopathy, even after adjustment for disease severity and other confounders [81,82].

The gut–liver axis has been proposed as one potential mechanism underlying these clinical associations. Suppression of gastric acidity promotes the overgrowth of *Enterococcus* species, normally constrained by gastric acid [33]. In murine models of chronic alcohol exposure, PPI administration resulted in the expansion of cytolysin-producing *Enterococcus faecalis*, increased intestinal permeability, and enhanced translocation of viable bacteria and bacterial products to the liver. Once in the liver, *Enterococci* and their components activate Toll-like receptors (TLR), particularly TLR2 and TLR4 on Kupffer cells, triggering inflammatory signaling pathways and increasing production of cytokines such as IL-1β, thereby amplifying hepatic inflammation and hepatocellular injury [33]. Genetic or pharmacological targeting of *E. faecalis* cytolysin, as well as bacteriophage-mediated depletion of cytolytic *Enterococci*, attenuated liver injury in alcohol-fed mice, providing causal evidence for this pathway [95].

However, findings from broader cirrhosis populations have not been uniform. In a multicenter cohort of 1160 patients with cirrhosis, PPI use was not independently associated with either minimal hepatic encephalopathy at baseline or the subsequent development of overt hepatic encephalopathy (sHR 1.13, 95% CI 0.81–1.59). Although this cohort was not restricted to alcohol-associated cirrhosis, these findings highlight the uncertainty surrounding the independent contribution of PPI exposure to hepatic encephalopathy risk [96].

Taken together, these experimental findings provide mechanistic plausibility for a role of PPI-associated microbial alterations in alcohol-related liver injury. However, whether these mechanisms directly mediate the adverse clinical outcomes observed in patients with ALD remains to be established.

### 4.4. Gastric Cancer

In recent decades, accumulating epidemiological evidence has suggested an association between long-term PPI use and an increased risk of gastric cancer. Recent meta-analyses consistently report relative risks ranging from approximately 1.3 to 2.9 across different populations, with risk increasing with duration and cumulative exposure [84,85]. Population-based cohort studies indicate that this association persists even after *Helicobacter pylori* eradication, with long-term PPI therapy resulting in a dose-dependent increase in gastric cancer risk, whereas H2 receptor antagonists do not show a comparable effect [86]. However, not all large-scale epidemiological studies have confirmed this association. A recent population-based study including 17,232 cases of non-cardial gastric adenocarcinoma and 172,297 controls across five Nordic countries found no association between long-term PPI use and gastric adenocarcinoma after adjustment for relevant confounders (adjusted OR 1.01, 95% CI 0.96–1.07). Notably, adjustment for *H. pylori*-related variables substantially attenuated the crude association, illustrating the potential impact of confounding and protopathic bias in this setting [97]. Overall, although several studies have reported consistent associations across populations and exposure durations, residual confounding, confounding by indication, and protopathic bias remain important limitations, and the available observational evidence does not establish a causal relationship between proton pump inhibitors use and gastric cancer [85,87].

Mechanistic studies nevertheless provide biological plausibility for a potential association. Profound and sustained acid suppression induces chronic hypergastrinemia, which acts as a trophic stimulus in the gastric mucosa and promotes enterochromaffin-like cell proliferation and mucosal hyperplasia [38]. Gastrin and its receptor are expressed not only in normal oxyntic mucosa but also in premalignant lesions such as atrophic gastritis and intestinal metaplasia, as well as in intestinal-type gastric adenocarcinoma, suggesting a role for gastrin-driven signaling in gastric carcinogenesis [98]. In addition, hypochlorhydria alters the gastric microbiome, enabling bacterial overgrowth and increased formation of carcinogenic N-nitroso compounds, processes that may accelerate malignant transformation, particularly in the presence of *H. pylori*-induced mucosal damage [84,85]. Animal studies further support these mechanisms, as profound acid inhibition consistently induces gastric neoplasia across species, highlighting the importance of gastric acidity in maintaining epithelial homeostasis [84].

PPIs are therefore unlikely to act as direct carcinogens but might create a permissive microenvironment that amplifies established pathways of gastric carcinogenesis.

### 4.5. Colorectal Cancer

Unlike gastric cancer, the epidemiological association between PPI exposure and colorectal cancer (CRC) remains uncertain. Several meta-analyses and large observational studies have investigated this relationship without demonstrating a reproducible, statistically significant increase in CRC risk among PPI users compared with non-users or individuals receiving alternative acid-suppressive therapies [88,89,90,91]. For example, a meta-analysis including nine observational studies and more than one million participants found no statistically significant overall association between PPI use and CRC (pooled OR 1.26, 95% CI 0.90–1.73), although a weak association emerged when exposure was restricted to use exceeding five years [91].

From a mechanistic perspective, PPI-induced gastrointestinal oralization represents a compelling biological link between proton pump inhibitor exposure and CRC-associated dysbiosis, although this relationship has not yet been conclusively demonstrated. As previously discussed, PPI therapy increases the transmission and enrichment of oral-derived taxa within the gut microbiota [56,57]. This pattern substantially overlaps with CRC-associated fecal and mucosal microbiome signatures, which frequently include oral and periodontal taxa belonging to *Fusobacterium, Parvimonas*, *Peptostreptococcus*, *Porphyromonas*, and *Streptococcus* [99,100,101]. Several of these microorganisms, particularly *F. nucleatum*, have been implicated in colorectal cancer development and progression through their pro-inflammatory, pro-proliferative, and immunomodulatory properties [101,102].

PPI-induced loss of colonization resistance may therefore facilitate the establishment and persistence of an oral-derived, pro-carcinogenic microbial community. Although direct longitudinal confirmation is still required, the available evidence supports PPI-induced oralization as a biologically credible contributor to the emergence of CRC-associated microbial signatures.

## 5. Proton-Pump-Inhibitor-Associated Dysbiosis and Immunomodulatory Effects: Implications for Cancer Immunotherapy

In addition to their primary role in gastric acid suppression, PPIs exert immunomodulatory effects that may influence host defense and immune-mediated disease [3,60]. PPIs may influence immune homeostasis at multiple levels, affecting both adaptive and innate immune responses [3,60].

At the adaptive level, PPIs directly affect T-cell biology. On human CD4^+^ T cells, PPI exposure suppresses lymphocyte proliferation and downregulates the vacuolar hydrogen/potassium adenosine triphosphatase (H^+^-ATPase), which is involved in intracellular pH regulation and T-cell activation. These changes are associated with altered differentiation pathways, including reduced Th1 and regulatory T-cell (Treg) development together with relative expansion of Th17 responses, an immune profile linked to chronic inflammation and autoimmunity [3,103]. This Th17/Treg imbalance occurs within a broader pattern of immune dysregulation characterized by altered cytokine signaling pathways, with reduced production of mediators such as interleuchina 1β (IL-1β), interleuchina 6 (IL-6) and tumor necrosis factor alpha (TNF-α) and diminished generation of reactive oxygen species [104].

Innate immune functions may also be affected through multiple pathways. Reduced neutrophil chemotaxis, phagocytosis, oxidative burst and bactericidal activity have been reported following PPI exposure [79,80]. In addition, compound-specific studies suggest that the immunomodulatory effects of PPIs may extend to other innate immune compartments: reduced natural killer cell cytotoxicity has been reported with omeprazole and lansoprazole, whereas rabeprazole promoted a shift towards M2 macrophage polarization in experimental models [105,106,107]. Inhibition of lysosomal enzyme activity may further interfere with antigen processing and presentation, potentially affecting systemic immune surveillance [108].

Clinically, these immune alterations may have divergent consequences. Impaired host defense may partly explain the increased susceptibility to infections observed among PPIs users [79,80]. On the other hand, large nationwide cohort studies have associated long-term PPI exposure with an increased incidence of both organ-specific and systemic autoimmune diseases, including rheumatoid arthritis and autoimmune thyroid disease [109].

Within this broader clinical framework, particular attention has focused on cancer immunotherapy, as PPI-associated immune alterations may influence antitumor responses and the efficacy of immune checkpoint inhibitors. In particular, increasing evidence indicates that concomitant PPI use is associated with the reduced efficacy of immune checkpoint inhibitors (ICI) [110,111].

Meta-analyses across heterogeneous cancers have consistently identified a statistically significant association between PPI exposure during immunotherapy and poorer survival outcomes, with pooled hazard ratios for overall survival ranging from approximately 1.31 (95% CI, 1.19–1.44) to 1.40 (95% CI, 1.25–1.57) and for progression-free survival from 1.28 (95% CI, 1.15–1.42) to 1.34 (95% CI, 1.19–1.52) [111,112,113]. The magnitude of this association varies according to tumor type, with the most consistent negative effects observed in non-small-cell lung cancer and urothelial carcinoma, whereas melanoma, renal cell carcinoma and hepatocellular carcinoma show more heterogeneous findings [108,113,114]. Timing and cumulative exposure also appear relevant: PPI use within roughly 30 to 60 days of ICI initiation is associated with the greatest reductions in survival, and increasing cumulative exposure correlates with poorer outcomes [112,115].

Part of this effect is thought to be mediated by PPI-induced dysbiosis, characterized by reduced microbial diversity and depletion of taxa associated with favorable responses to ICI therapy [116,117,118], including *Akkermansia muciniphila*, *Faecalibacterium prausnitzii* and members of the *Ruminococcaceae* and *Lachnospiraceae* families, associated with the expansion of oral and opportunistic bacteria [119,120,121]. By disrupting microbiota-derived immune signals, including short-chain fatty acids such as acetate, propionate, and butyrate, this dysbiotic state may compromise CD8^+^ T-cell activation and the expansion of antitumor effector cells required for effective checkpoint blockade [122,123].

Taken together, these findings identify PPI exposure as a potentially modifiable clinical factor associated with immune checkpoint inhibitor outcomes. However, the current evidence does not establish whether this association is directly mediated by microbiota alterations, direct immunomodulatory effects, residual confounding, or a combination of these factors. These data nevertheless support the careful reassessment of PPI therapy indication in patients undergoing cancer immunotherapy.

## 6. Role of Probiotics During PPI Therapy

Taken together, the evidence reviewed above identifies the gut microbiota not only as a potential mediator of PPI-associated adverse outcomes, but also as a promising therapeutic target. Microbiota-directed interventions may therefore provide an opportunity to prevent or mitigate the clinical consequences associated with PPI-induced dysbiosis.

Available strategies range from dietary interventions, prebiotics, probiotics, synbiotics and targeted non-absorbable antibiotics to more innovative approaches such as fecal microbiota transplantation, engineered bacteria and phage therapy [124]. However, among these strategies, probiotics are the most extensively investigated in the context of PPI-associated microbiota alterations and currently represent the only approaches supported by preliminary clinical evidence, although such evidence remains limited and heterogeneous [3,16]. Conversely, evidence for dietary interventions, prebiotics, postbiotics, or other microbiota-modulating strategies specifically aimed at preventing or reversing PPI-induced dysbiosis remains very limited.

Probiotics, defined as live microorganisms that confer health benefits when administered in adequate amounts [125], have been proposed as a strategy to counteract the previously described PPI-induced alterations in the gut microbiota, potentially restoring microbial homeostasis and limiting the overgrowth of opportunistic pathogens.

Interventional studies conducted in this setting have followed two complementary lines of investigation, assessing either microbiome-level endpoints or clinically relevant PPI-associated complications.

At the microbiome level, interventional studies have explored whether probiotic supplementation can attenuate PPI-associated microbial perturbations [58,126]. In a randomized, double-blind pilot study, 30 healthy adults received either omeprazole 20 mg/day or placebo for six weeks, with all participants receiving a high-dose multi-strain probiotic during the final four weeks. Longitudinal shotgun metagenomic and untargeted metabolomic analyses indicated that probiotic supplementation partially attenuated selected PPI-associated microbial and metabolic alterations [58]. Conversely, Horvath et al. evaluated a three-month, open-label intervention with a lower-dose, multistrain symbiotic in 36 clinically heterogeneous patients receiving continuous PPI therapy for at least six months. The intervention did not significantly alter overall microbiota composition or alpha diversity, producing only limited genus-level shifts, including reduced *Stomatobaculum* and increased *Bacillus* abundance [126]. However, the available studies were not designed to directly compare different timings of probiotic supplementation, and current evidence is therefore insufficient to determine whether the timing of administration influences its efficacy during PPI therapy. Further studies specifically addressing this question are needed.

Beyond these microbiome-level endpoints, probiotics have also been directly evaluated for their ability to prevent clinically relevant PPI-associated complications, particularly SIBO and *Clostridioides difficile* infection [127,128].

In a randomized study of 128 children with GERD, concomitant administration of *Lactobacillus reuteri DSM 17938* during 12 weeks of PPI therapy was associated with a markedly lower prevalence of SIBO, assessed by glucose hydrogen breath testing, compared with PPI therapy alone (6.2% vs. 56.2%; *p* < 0.001) [127]. Evidence regarding CDI prevention is less conclusive: in a recent retrospective study, *Lactobacillus reuteri DSM 17938* co-administration was associated with a numerically lower incidence of CDI among esomeprazole users, although the difference did not reach statistical significance (3.8% vs. 10.4%; *p* = 0.060) [128].

Importantly, the effects of probiotics are strain- and formulation-specific, and findings obtained with a given strain or multi-strain preparation cannot be directly extrapolated to other probiotic formulations. The heterogeneity of the available studies in terms of probiotic strains, combinations, doses, treatment duration, and timing of supplementation further limits direct comparisons across studies and precludes conclusions regarding probiotics as a single therapeutic category [129].

However, despite these potential benefits, safety concerns remain. Cases of fungemia and bacteremia have been reported in association with long-term probiotic use in high-risk patients [3]. Accordingly, the American Gastroenterological Association does not recommend the routine use of probiotics for infection prevention in chronic PPI users due to limited and heterogeneous evidence [16]. Further well-designed clinical studies are therefore needed to better define the efficacy, safety, and microbiome impact of probiotics during proton pump inhibitor therapy.

## 7. Conclusions

PPIs remain highly effective and often indispensable agents for the management of acid-related disorders. Nevertheless, their extensive and often inappropriate long-term use has raised safety concerns. Accumulating evidence indicates that PPIs profoundly influence the gastrointestinal microbiota through multiple mechanisms, including gastric acid suppression, impaired barrier function, and modulation of immune and metabolic pathways [126]. These changes promote oral-to-gut microbial translocation, reduce microbial diversity, and favor the expansion of potentially pathogenic taxa, with downstream clinical consequences such as small intestinal bacterial overgrowth, enteric infections, and alterations in the gut–liver axis. Emerging data further suggest that PPI-induced dysbiosis may have broader implications, including effects on carcinogenesis and reduced efficacy of immune checkpoint inhibitors.

Despite the biological plausibility of these associations, most clinical evidence remains observational and is therefore vulnerable to confounding by indication, underlying comorbidities and concomitant medication use. This is particularly relevant because PPIs are frequently prescribed in the context of polypharmacy, and several commonly used medications may independently alter gut microbiota composition and function, making it difficult to disentangle PPI-specific effects in real-world studies. Additional heterogeneity arises from differences in PPI doses and durations and in the definitions of microbiome-related outcomes.

Although microbiota-directed interventions, particularly probiotics, represent a promising strategy, current evidence remains insufficient to support their routine use. Future studies should adopt prospective longitudinal designs with microbiome sampling before PPI initiation, during treatment, and after discontinuation in order to characterize the temporal dynamics and potential reversibility of PPI-associated microbial changes. PPI exposure should be standardized and reported in detail, including the specific compound, dose, treatment duration, timing, and cumulative exposure. Studies should also carefully account for major microbiome-modifying factors, including diet, underlying comorbidities, antibiotic exposure, and concomitant medications. Integration of taxonomic profiling with functional metagenomics, metabolomics, and clinically relevant outcomes may help distinguish compositional changes from biologically meaningful functional alterations. Finally, adequately powered interventional studies and mediation analyses are needed to determine whether microbiota alterations contribute directly to PPI-associated clinical outcomes or primarily represent an accompanying marker of drug exposure and underlying disease.

In this context, careful, indication-based prescribing of PPIs is essential to minimize unnecessary exposure and limit microbiota alterations and their clinical consequences.

## Data Availability

No new data were created or analyzed in this study. Data sharing is not applicable to this article.
